# Bayesian models for comparative analysis integrating phylogenetic uncertainty

**DOI:** 10.1186/1471-2148-12-102

**Published:** 2012-06-28

**Authors:** Pierre de Villemereuil, Jessie A Wells, Robert D Edwards, Simon P Blomberg

**Affiliations:** 1 School of Biological Sciences, University of Queensland, Brisbane, 4072 Queensland, Australia; 2 Department of Biology, École Normale Supérieure, , 45 rue d’Ulm, 75005 Paris, France

## Abstract

**Background:**

Uncertainty in comparative analyses can come from at least two sources: a) phylogenetic uncertainty in the tree topology or branch lengths, and b) uncertainty due to intraspecific variation in trait values, either due to measurement error or natural individual variation. Most phylogenetic comparative methods do not account for such uncertainties. Not accounting for these sources of uncertainty leads to false perceptions of precision (confidence intervals will be too narrow) and inflated significance in hypothesis testing (e.g. p-values will be too small). Although there is some application-specific software for fitting Bayesian models accounting for phylogenetic error, more general and flexible software is desirable.

**Methods:**

We developed models to directly incorporate phylogenetic uncertainty into a range of analyses that biologists commonly perform, using a Bayesian framework and Markov Chain Monte Carlo analyses.

**Results:**

We demonstrate applications in linear regression, quantification of phylogenetic signal, and measurement error models. Phylogenetic uncertainty was incorporated by applying a prior distribution for the phylogeny, where this distribution consisted of the posterior tree sets from Bayesian phylogenetic tree estimation programs. The models were analysed using simulated data sets, and applied to a real data set on plant traits, from rainforest plant species in Northern Australia. Analyses were performed using the free and open source software OpenBUGS and JAGS.

**Conclusions:**

Incorporating phylogenetic uncertainty through an empirical prior distribution of trees leads to more precise estimation of regression model parameters than using a single consensus tree and enables a more realistic estimation of confidence intervals. In addition, models incorporating measurement errors and/or individual variation, in one or both variables, are easily formulated in the Bayesian framework. We show that BUGS is a useful, flexible general purpose tool for phylogenetic comparative analyses, particularly for modelling in the face of phylogenetic uncertainty and accounting for measurement error or individual variation in explanatory variables. Code for all models is provided in the BUGS model description language.

## Background

Comparative analysis is a central tool in evolutionary biology and ecology: if we wish to understand the co-evolution of traits and their relationships with their environment, comparisons among species can identify relationships among traits and environmental variables that signify underlying evolutionary or ecological processes. The use of comparative studies allows biologists to address important concepts like adaptation [1,2], evolution of behavioural and morphological traits [3-5], allometry [6-8] or basal metabolism rate evolution, e.g. [9,10].

**Figure 1:**
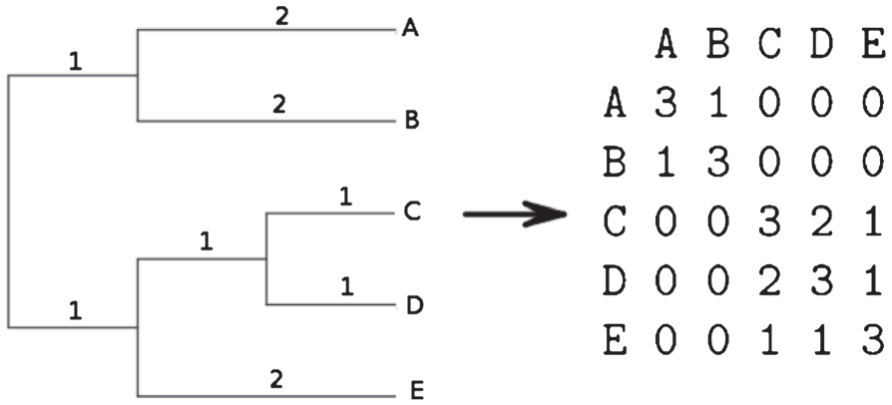
**Transformation from a phylogenetic tree to a variance-covariance matrix under the Brownian Motion (BM) model: the variance is set to be the branch length from the root to the tip.** The covariance is the branch length from the root to the most recent common ancestor.

Often, comparative studies summarise relationships using correlation or regression coefficients. Such analyses require special tools to take into account the phylogeny of species, as their shared evolutionary histories lead to phylogenetic structure in the data (a specific form of non-independence of data) [11]. We therefore need to take the phylogeny into account. The most common approach has been to apply the method of independent contrasts [11], or alternatively, to model the data using a multivariate normal distribution which has a covariance structure derived from the phylogenetic tree. For a specific tree with computed (but possibly arbitrary) branch lengths and with a model of character evolution (often Brownian Motion (BM) [11]), one can derive a variance-covariance matrix, *Σ*[12]. In the case of the BM model (which we assume here and throughout the rest of the paper) the covariances are proportional to the shared branch length from the most recent common ancestor of each pair of taxa to the root (Figure 1).

We can fit a linear regression between data vector *Y * and an explanatory variable *X* using this variance-covariance matrix and the multivariate normal distribution:

(1) Y|X∼N(Xβ,Σ)

where *β* is a vector of regression coefficients. The multivariate distribution is used to model one variable for multiple tips on a tree, rather than to model multiple characters. Indeed, in this paper, we restrict the analysis to the simple case of regression of one explanatory variable (*X*) and one response variable (*Y *), however the models can easily be extended to the multiple regression case, and also to the multivariate response case.

This "phylogenetic" regression can easily be computed using Generalised Least Squares, GLS [13,14]. A problem with this method is that it depends on both the topology and branch lengths of the tree, which are assumed to be known without error [2]. Any phylogenetic tree that we estimate is unlikely to be an exact representation of the true phylogeny, due to bias or uncertainties from several sources, such as data sampling, sequence alignment, choice of models of sequence evolution, low resolution such that many topologies appear similarly probable, homoplasy or artefacts such as long-branch attraction [1,15,16].

Ideally, we should directly incorporate phylogenetic uncertainty into our models, because this will give us a more "honest" analysis, with correct p-values and estimated parameter distributions that more fully represent the current state of our knowledge. To assume no phylogenetic nor measurement uncertainty may lead to bias and may severely overestimate out confidence in the conclusions. Since it can be difficult to derive an accurate tree, comparative studies should allow for uncertainty in the phylogeny [1]. Many authors have suggested methods for dealing with this uncertainty (e.g. [13-15,17-22]). Several have proposed frequentist methods to model phylogenetic uncertainty, (though see [23,24] for a critique), while [25,26] proposed the incorporation of phylogenetic uncertainty in Bayesian comparative analyses through the use of prior distributions on phylogenetic trees. In this paper, we use this idea to develop some common models using the freely available OpenBUGS program [27]. We use this program because its syntax is easily understood and modifiable, and (along with the almost identical WinBUGS; [27,28]) is the most commonly used software for Bayesian analysis within the wider statistical community (although it is not used for inferring phylogenies). A full tutorial on the use of OpenBUGS is beyond the scope of the present paper. Readers who are interested in learning how to use OpenBUGS as a general statistical analysis tool are encouraged to consult the extensive manual that is part of OpenBUGS, the OpenBUGS web site ( http://www.openbugs.info), or introductory books such as [29,30].

Bayesian statistics is based on Bayes’ Theorem, which can be expressed by the equation:

(2) $$P(B|A)=\frac{P(A|B)P(B)}{P(A)}$$

where *A* and *B* are two events, and *P*(*X*) is the probability of event *X*. This relationship makes it possible to find the probability of *B* given *A*, if you have information on the probability of *A* given *B* and the probabilities of *A* and *B* (knowledge of *P*(*A*) is usually unnecessary as it is simply a normalising constant). In Bayesian statistical inference, *B* stands for the parameters to be estimated and *A* stands for the observed data. When a set of data is observed, we can apply a model and construct a likelihood function for the probability of observing the data *y*, given the parameters *θ*: *P*(*y*|*θ*). However, we wish to know the probability of a hypothesis (or parameter values) given the observed data *P*(*θ*|*y*). To find these values we apply Bayes’ theorem, combining the likelihood with a set of prior distributions for the parameters, and obtaining the posterior probability distributions of the parameters, given the data. The prior distributions express any knowledge or ignorance about the model parameters before the data are collected. Such priors can be based on earlier data sets, an expression of existing knowledge, or can be minimally informative, for example as diffuse distributions over all logical possibilities. For simplicity, since the probability of the data *P*(*y*) is a normalising constant, Bayes’ theorem can be written as:

(3) f(θ|y)∝L(y|θ)p(θ)

*f*(*θ*|*y*) is called the posterior distribution of the estimated parameter, *L*(*y*|*θ*) is the likelihood of the data, given the parameters, and *p*(*θ*) is the prior distribution of the parameters *θ*[31-34]. One advantage of this framework is that it allows us to incorporate a distribution for the phylogenetic tree (in terms of a distribution of variance-covariance matrices *Σ*) and then to integrate over this distribution to take into account all the possible trees:

(4) f(θ,y)=p(θ)∫L(y|θ,Σ)p(Σ|θ)dΣ

where *Σ* stands for the variance-covariance matrix (as in Eqn. 1) associated with each tree. Thus, one can calculate the likelihood of the data *L*(*y*|*θΣ*) and then incorporate the uncertainty in the phylogeny using the distribution of *Σp*(*Σ*|*θ*). An informative distribution of the phylogeny can be defined by using an empirical distribution of trees [25,26]. Since Bayesian tools already exist for phylogenetic tree estimation, we can use the posterior sample of trees that is generated by such tools as BEAST [35] or MrBayes [36] as the prior distribution for our analysis. This approach has been used in the program BayesTraits [37], which can fit multiple regression models to multivariate Normal trait data.

Here, we show how phylogenetic uncertainty can be incorporated in many of the models that biologists commonly employ. BUGS code for each model is provided in an Additional file 1. With some minor changes, these scripts can also be run using the program JAGS [38]. We explore the behaviour of the models using simulated data sets, and illustrate their application to two real data sets of plant traits. Finally, we discuss the performance and relevance of this approach, and possibilities for extensions.

## Results

### Data analysis & results

#### Linear regression model with simulated data

Using this simple model, we will illustrate the value of using empirical tree distributions for comparative analysis. As an example of frequentist analysis tools, we will use the R function gls() from the nlme package [39], which enables linear regressions to incorporate a covariance structure to the residuals. Since we are performing a regression with only one predictor variable, we redefine the regression as $E(Y{|}_{X,\theta })=\beta_{0}1+\beta_{1}X$, where 1 is a vector with all components set to 1.

**Figure 2:**
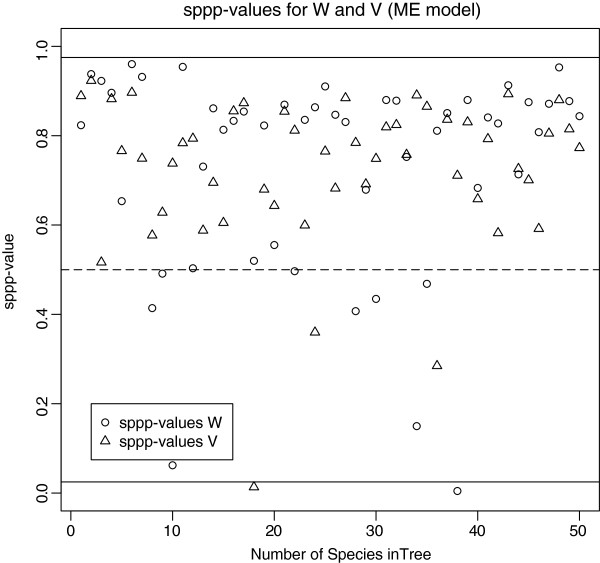
**Distribution of the individual measurements sppp-values for the Measurement Error (ME) model.** Solid lines are the 2.5% and 97.5% limits, dashed line is for 50%.

We used a set of 100 trees from the posterior distribution of a phylogeny of rainforest plant species generated using BEAST in an analysis of trnL-F chloroplast sequences (J. Wells, unpubl.). We chose 100 trees as being a reasonable compromise between the sampling error of the trees and computational convenience (see the technical discussion for details of memory usage and computation times for up to 10,000 trees). For simulations, we selected one tree to be the "correct" tree (Figure 2) and simulated data sets of trait values along that tree for 50 species, from a linear regression model with regression parameters *β*_0_=5, *β*_1_=2 and residual variance *σ*of 2, 5, 10 or 15. We then used the whole set of trees from BEAST to construct a strict consensus tree. We used the function gls() from the package nlme for R to fit the GLS linear regression using the consensus tree, then again, using the correct tree. Finally, we fitted the linear regression using our Bayesian models with either the consensus tree (One Tree, or OT) or all 100 trees (AT) (Table 1. We replicated this procedure for 3,000 simulations. Distribution of the estimates for *β*_0_, *β*_1_ and *σ* showed no systemic bias for the first parameters (Figure 2). However, methods using a single consensus tree (GLS or Bayesian OT model) overestimated residual variance *σ*, whereas GLS with the true phylogenetic tree and the Bayesian model incorporating phylogenetic uncertainty (Bayesian AT) are both more accurate. This has consequences for the precision of *β*_0_ and *β*_1_: estimates based on true GLS and Bayesian AT are more precise than estimates based on methods using the consensus tree. When comparing the average widths of 95% confidence or credible intervals, as a measure of the uncertainty, we see that there is higher uncertainty when using methods based on the single consensus tree. Even though these consensus-tree based estimates are more uncertain, we also see that they yield higher Type 1 error rates associated to confidence or credible intervals (i.e. proportion of times that the estimated interval does not contain the true parameter value). These error rates are approximately twice as high as expected for the slope *β*_1_ (i.e. roughly 10% error, rather than the nominal 5%). This situation of anti-conservative coverage despite higher uncertainty, is likely to originate from the lower precision of estimates based on single consensus trees. Note that credible intervals based on the Bayesian AT method can yield anti-conservative coverages as well (Table 1, Bayes AT). However, in this case, the deviations from the expected rate (5%) are relatively small.

**Table 1:** Mean 95% confidence or credible interval size with associated Type I error rate (true value outside interval), based on 3,000 simulated datasets


**True *σ* **	**Estimation method**	** *β* **_0_		** *β* **_1_		** *σ* **	
		**CI size**	**Error rate**	**CI size**	**Error rate**	**CI size**	**Error rate**
2	Real GLS	6.12	0.002^∗^	0.23	0.121^∗^	1.74	1.000^∗^
	True GLS	3.39	0.044	0.20	0.058	0.83	0.041
	Bayes AT	3.26	0.057	0.20	0.063^∗^	0.86	0.037^∗^
	Bayes OT	5.99	0.002^∗^	0.22	0.131^∗^	1.67	1.000^∗^
5	Real GLS	15.34	0.003^∗^	0.57	0.125^∗^	4.37	1.000^∗^
	True GLS	8.50	0.057	0.49	0.059	2.07	0.050
	Bayes AT	8.11	0.070^∗^	0.49	0.066^∗^	2.14	0.045
	Bayes OT	15.02	0.003^∗^	0.56	0.133^∗^	4.18	1.000^∗^
10	Real GLS	30.43	0.003^∗^	1.14	0.115^∗^	8.66	1.000^∗^
	True GLS	16.93	0.053	0.99	0.044	4.13	0.056
	Bayes AT	16.16	0.069^∗^	0.99	0.056	4.27	0.051
	Bayes OT	29.78	0.004^∗^	1.11	0.123^∗^	8.30	1.000^∗^
15	Real GLS	45.72	0.002^∗^	1.70	0.119^∗^	13.01	1.000^∗^
	True GLS	25.36	0.051	1.46	0.054	6.19	0.051
	Bayes AT	24.18	0.070^∗^	1.46	0.062^∗^	6.38	0.048
	Bayes OT	44.72	0.003^∗^	1.66	0.128^∗^	12.47	1.000^∗^

Simulated date were *β*_0_=5, *β*_1_=2 and several levels for *σ* parameter (see first column). Real GLS : Frequentist least-square using consensus tree; True GLS : Frequentist least-square using true phylogenetic tree; Bayes AT: Bayesian method using 100 trees (estimated from a real dataset) including the true tree; Bayes OT: Bayesian method using consensus tree. Marked error rate (^∗^) are significantly different from expected 5% error rate (Binomial test, *p*<0.01).

#### Linear regression model with real data

To check the behaviour of our models with real data, we used real trait measurements (stem-tissue density and leaf-tissue density) for seedlings of the species in the rainforest phylogeny mentioned above (J. Wells, unpubl.). We modelled this data set using the simple Linear Regression model in its frequentist (GLS) and Bayesian form, and a regression model incorporating Pagel’s *λ* as an estimator of phylogenetic signal in the trait, beyond the structure embodied in the variance covariance matrix *Σ*(PL; Table 2). We fitted the frequentist version of Pagel’s lambda regression using the gls() function in the nlme[39] and ape[40] package for R. For the Linear Regression model, we observed a strong disagreement between the GLS method and the Bayesian model, especially concerning *β*_1_. This probably resulted from the consensus tree being a poor summary of the true tree, which is supported by the low estimation of *λ* by the GLS Phylogenetic Signal model.

**Table 2:** **Results of a linear regression applied to real trait data (leaf tissue density and stem density for 200 rainforest plant species), with (PL) or without (LR) an extra parameter  *λ* to quantify the strength of phylogenetic signal in the Y-axis trait**


	**LR model**		**PL model**	
**Parameter**	**GLS**	**Bayesian (AT)**	**GLS**	**Bayesian (AT)**
*β*_0_	-1.07 (0.47)	-0.70 (0.31)	-0.75 (0.14)	-0.50 (0.11)
*β*_1_	0.31 (0.13)	0.62 (0.10)	0.55 (0.11)	0.58 (0.11)
*λ*	—	—	0.24 (-0.12, 0.63)	0.82 (0.13)
*σ*	1.18	0.70 (0.074)	0.33	0.77 (0.095)
*ppp-value*	—	0.545	—	0.9974

Values are: *mean (standard deviation)*, except for *λ*, which is *mean (95% Confidence limits)*. The value *σ*for the gls() function is the residual standard error.

**Figure 3:**
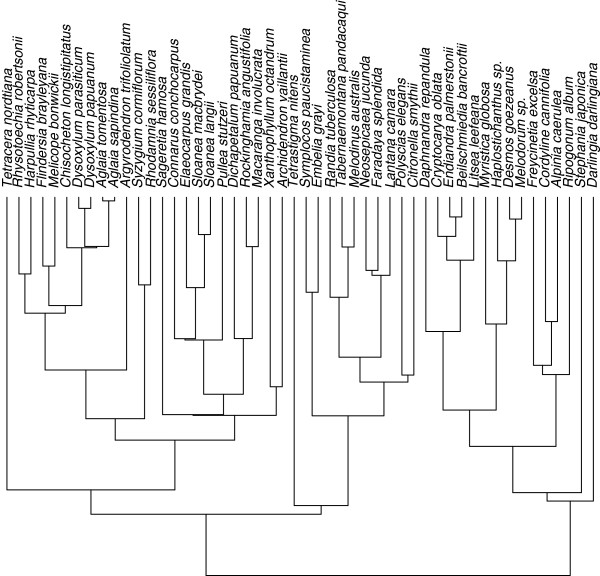
**Phylogeny of the "correct" tree used for simulated data.** Number of species: 50.

The phylogenetic signal strength is estimated in the response variable *Y * in the Phylogenetic Signal model, and allows for some mis-specification in phylogeny branch lengths. Hence, GLS and the Bayesian model are more in agreement regarding regression parameters *β*_0_and *β*_1_ compared to the Linear Regression model discussed above. However, although the ppp-value for the Linear Regression model is good (0.545; see Figure 3), the ppp-value for the Phylogenetic Signal model is 0.9974 which suggests a problematic overdispersion of the replicated residuals compared to the real data residuals. This is probably because the Phylogenetic Signal model underestimates the phylogenetic signal, since fixing *λ* to 1 when simulating replicates brings the ppp-value down to 0.75. The correlation structure of the data appears to be sufficiently strong, that it can be well represented by the variance-covariance matrix in the Linear Regression model, and it does not improve the model goodness-of-fit to further estimate an additional parameter for the phylogenetic signal(*λ*). This is an example of how ppp-values can help to detect failures in models. The 95% credible interval for *β*_1_ is [0.42;0.82]: the slope is clearly positive. We conclude that the density of seedling leaf tissue scales positively with the density of the stem, as predicted if species with higher density (and thus better protected) seedling leaves, also invest in more robust stems.

#### Measurement error model

**Figure 4:**
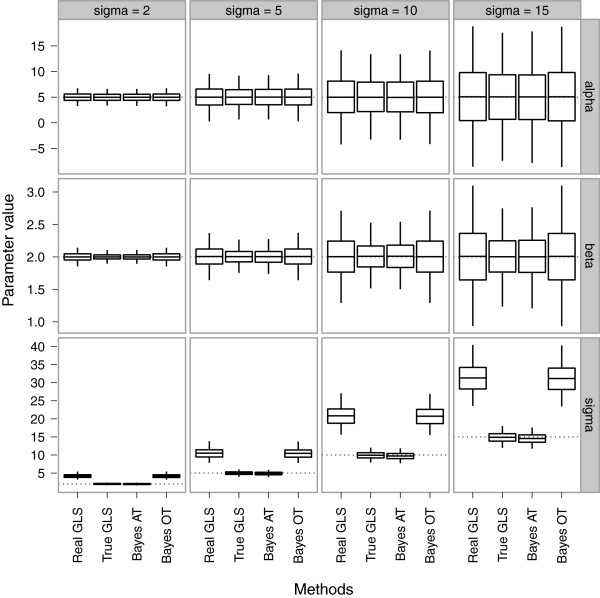
**Distribution of estimates for *β***_** *0* **_**, *β* **_***1 ***_**and  *σ* for simulated data based on 3,000 simulations.** Simulated values for *β*_0_ and *β*_1_ are 5 and 2 (dotted lines). Simulated values for *σ* are 2, 5, 10 and 15 (dotted lines). Boxes represent 25% and 75% quantiles and the middle line represents the mean. Whiskers show 2.5% (bottom) and 97.5% (top) quantiles.

The previous example analysed data for which there was only one datum per species. The data set was actually a subset of a larger data set with replicated measurements for each species, in order to model variation among individuals and/or variation due to technical measurement error (conceived broadly as "Measurement Error"). Instead of a vector of species measurements as for the Linear Regression model, we constructed matrices of individual measurements (columns), for each species (rows). Note that, in the Measurement Error model, matrices of measurements *W * and *V * correspond to traits *X* and *Y *, respectively. Results for fitting the Measurement Error model are presented in Table 3. The Measurement Error model yields different results compared to the earlier Linear Regression model, with slightly higher spread of the posterior distributions, as is expected when changing from a model where *X* is a fixed predictor to a model where both *X* and *Y * are random variables. There is still strong evidence for the existence of a positive slope, with a 95% credible interval for *β*_1_ of [0.46, 0.99]. In posterior checks, the ppp-value for estimates of the species-level values was acceptable. However, the distribution of sppp-values based on the individual measurements showed a slight but consistent overdispersion of the replicates compared to the real data distribution (see Figure 4). This suggests two possibilities: a) a covariance structure exists for individual measurements within a species, for example as may arise from population genetic structuring, or b) *σ*_*V*_ and *σ*_*W*_ were not constant across species, for example if some species contain a wider range of genetic variants or show higher phenotypic plasticity in the expression of a trait.

**Table 3:** Results for the Measurement Error (ME) model applied to real trait data (leaf tissue density and stem density for 200 rainforest plant species)


**Parameters**	**ME model**	**LR model**
*β*_0_	-0.59 (0.37)	-0.70 (0.31)
*β*_1_	0.72 (0.13)	0.62 (0.10)
*σ*_*R*_	0.59 (0.078)	0.70 (0.074)
*σ*_*V*_	0.15 (0.0082)	—
*σ*_*W*_	0.14 (0.00074)	—
*ppp-value*	0.323	0.545

Values are: *mean (standard deviation)*. For comparison, estimates from the Linear Regression (LR) model are given.

#### Computational performance

**Figure 5:**
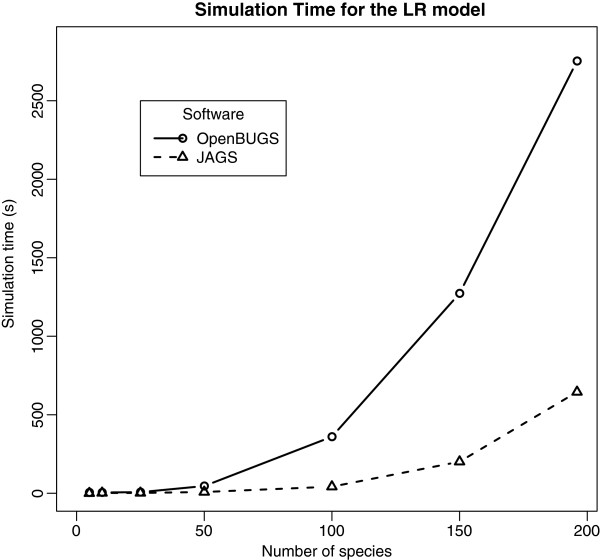
**Simulation time as a function of the number of species for the Linear Regression (LR) model (with 100 trees). ***Solid*: Running in OpenBUGS, *dashed*: running in JAGS.

**Figure 6:**
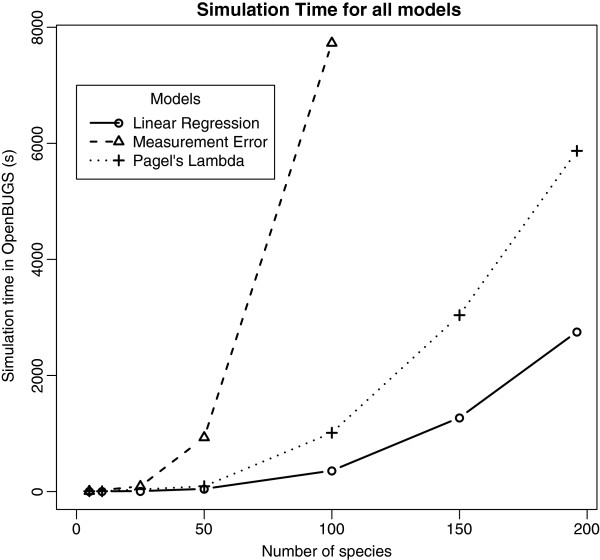
**Simulation time as a function of the number of species for all models in OpenBUGS with 100 trees. ***Solid*: Linear Regression (LR), *dashed*: Measurement Error model (ME), *dotted*: Phylogenetic Signal model using Pagel’s Lambda (PL).

**Figure 7:**
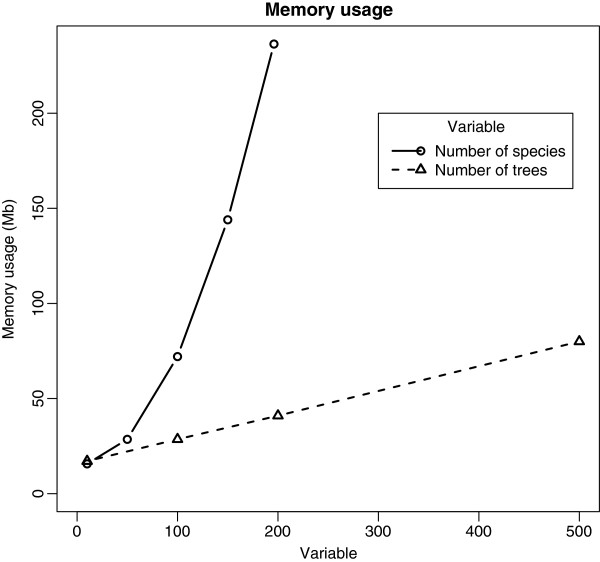
**Memory usage in OpenBUGS for the linear regression model. ***Solid*: as a function of the number of species with 100 trees, *dashed*: as a function of the number of trees with 50 species.

We performed an analysis of simulation time and memory use for our models. The two main factors that may influence simulation performance are the number of species *N* and the number of trees *K*. However, *K* has only a minor effect on simulation time (from 30s for *K* = 1 to 53s for *K* = 500 for Linear Regression model and 10,000 iterations), because introducing a new tree into the data has almost no impact on the numbers of parameters in the BUGS model. Figure 5 shows the relation between simulation time and *N* for the Linear Regression model: JAGS performs better than OpenBUGS (due to computational issues we explore in the Discussion). Figure 6 shows the simulation time as a function of *N* for all models, using OpenBUGS and an empirical prior distribution (*K* = 100): the simulation time for the Measurement Error model is very sensitive to *N* due to large matrices of individual measurements and it failed to run after compilation for N≥100 (although the model ran in JAGS). The Phylogenetic Signal model is also sensitive to *N* because of the need to transform the variance-covariance matrix *N*×*N*. For memory usage (Figure 7), once again the number of species is more important than the number of trees, which is understandable since the largest variable is the *N*×*N*×*K* array of inverses of variance-covariance matrices.

### Discussion

**Figure 8:**
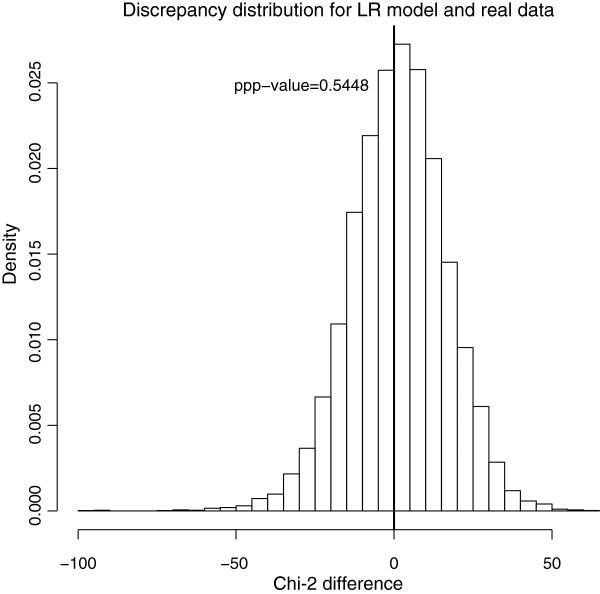
Distribution of the *χ*^2^-like discrepancy difference $D(y_{l}^{\mathrm{rep}},\theta_{l})-D(y,\theta_{l})$ for linear regression model with empirical prior and real data. The ppp-value is the proportion of values above zero.

We have shown that Bayesian methods for phylogenetic comparative analysis are easy to implement in the BUGS language, often only requiring several lines of code. This puts Bayesian methods within the reach of all researchers who wish to adopt the Bayesian mode of inference for phylogenetic comparative analyses. Since Bayesian methods provide a natural way of incorporating identifiable sources of error into an analysis, we believe Bayesian methods should become more common in comparative studies. We emphasise that failing to account for obvious sources of uncertainty in a statistical analysis is very likely to lead to more imprecise estimates (Figure 8) and illusory confidence intervals (Table 1).

Bayesian methods allow the modelling of multiple sources of uncertainty through the explicit use of prior distributions on model parameters. Because they require the quantitative representation of parameter uncertainty, Bayesian methods offer an excellent framework for the integrated analysis of comparative data that contain several sources of uncertainty, such as phylogenetic error and measurement error. Allowing for several sources of error in a frequentist analysis is more difficult, although [11,17,41] proposed a method for correcting for phylogenetic uncertainty, using a sample of trees from a phylogenetic bootstrap analysis. A bootstrap sample can be used to estimate the sampling distribution of a statistic, but the Bayesian approach results in the full posterior distribution of the statistic, conditional on the data. It is not clear how to interpret such a bootstrap sample as a posterior distribution of trees, as there is no notion of a prior on the trees in a frequentist bootstrap analysis. There is a Bayesian version of the bootstrap which estimates the posterior distribution of a statistic [42], however most recent research has concentrated on MCMC estimation methods. Lo [43] found that the ordinary bootstrap and the Bayesian Bootstrap have equivalent large-sample properties, and the Bayesian bootstrap sample can be considered as a posterior distribution if we assume a "flat" Dirichlet process prior. We are not aware of any applications of the Bayesian bootstrap to phylogenetic data, although [44] have used it for comparing protein sequences.

Although we have only explored three possible phylogenetic comparative models here, it is clear that the BUGS formalism is likely to be able to represent almost any reasonable Bayesian comparative model. Further, researchers can use our programs as building blocks to modify and combine analyses. For example, it is easy to combine the Measurement Error and Phylogenetic Signal models to form a measurement error model which simultaneously estimates phylogenetic signal.

We have demonstrated how to model phylogenetic uncertainty using an empirical prior set of trees derived from the output of Bayesian phylogenetic tree estimation programs. Use of this empirical prior is most attractive, because our simulations show that the estimates of regression coefficients are more precise and unbiased for residual variance (Figure 8). We now elaborate on the above issues.

#### Technical choices

Both OpenBUGS and JAGS use Markov Chain Monte Carlo (MCMC) algorithms and are based on the BUGS syntax. JAGS has a more flexible interpretation of the BUGS syntax than OpenBUGS, allowing the simplification of some parts of the computation (see Additional file 1), and making JAGS faster than OpenBUGS for our particular models. One last difference is that OpenBUGS has a Graphical User Interface (GUI). Thus, if a GUI is required, one might prefer to use OpenBUGS. Conversely, if one has a data set containing many species, JAGS may be a better choice. JAGS may fail if the variance covariance matrix is difficult to invert (probably due to problems if the matrix is only borderline positive-definite), however, this issue can be solved by setting a very small initial value for the precision tau (see Additional file 1), such as 0.001.

In this study, we used a relatively small number of sampled trees (usually 100) for computational convenience. However, for a real study, using a large number of trees is expected to better represent their true probability distribution, and hence decreases the Monte Carlo error and the impact of any very unlikely tree. We have seen that the number of trees *K* has a small impact on simulation time and a linear impact on memory usage. However, it still has an important impact in data handling (on number of matrices to be generated and inverted) and data loading/computation time in OpenBUGS and JAGS. Therefore, using more than 10,000 trees can become problematic (for 50 species, it will represent around 1.5Gb to compute, load and handle in memory). When the available set of trees is larger than the number K that we wish to sample for our empirical prior, we need to decide how to sample K trees from this larger set. The most straightforward way is to take a random sample of *K* trees directly from the empirical distribution at hand. This is relevant if *K* is large enough, but if *K* is small, the sampling error might be important and our sample might incorporate some trees with very low probability that would have an important impact in the comparative analysis. One way to avoid this may be to reject any sampled tree which has a posterior probability less than $\frac{1}{K}$, enabling the sample to be seen as a set of ’plausible’ trees at least partially covering the full tree distribution.

#### Issues & perspectives

For some data sets with a large number of species and a small number of trees (for example *N* = 150 and *K* = 100), the MCMC simulation may become "locked" on one specific tree (drawing it over and over in many iterations), instead of exploring the space of possible trees. Indeed, due to the relatively large number of species, there is a strong tendency to select one tree against the others: thus, the algorithm keeps rejecting any tree other than the one that fits the data the best. This might sound like a positive point, but we are interested in modelling uncertainty, not selecting *one* good tree. Consequently, one should interpret this behaviour as a sign that most of the trees in the sample are a very poor representation of the phylogenetic relationships, or that the sample of trees is simply too small. A way to solve this issue would be to add more trees to the sample, or, if this is not possible, to reduce the number of species that are included in the comparative analysis (for example to find a well-supported sub-tree within the full phylogeny, and perform the analysis on this set of species).

The results presented here all use the simple Brownian Motion (BM) model of character evolution, but one can use any other model in the process of computing variance-covariance matrices (e.g. models proposed by [14] or [57]). Our regression models focused only on linear relationships, but the Linear Regression model can easily be extended to non-linear relationships between *X* and *Y *. However, the multivariate normal distribution used to model the data would be difficult to replace by another one, because few continuous multivariate distributions are available (although for overdispersed data, one can use the heavy-tailed multivariate Student’s t in place of the normal [33, p.446]).

The Measurement Error model enables us to estimate the linear relation between two variables when both are random, and so we aim to estimate their joint variation rather than assigning a direction of prediction from an ’explanatory’ variable to a ’response’. It is also free from the need to assume that the error variances of X and Y are equal, or that the ratio of error variances equals the ratio of variances (as is required in Major Axis methods or Standardised Major Axis methods, see [45]). Also, this model offers several advances over existing methods for comparative analyses that incorporate variation below the species level: it enables consideration of phylogenetic uncertainty that is missing from several comparative methods that include individual variation, such as Felsenstein’s [55] Independent Contrasts method. A possible extension of our Measurement Error model would be to estimate the intraspecific variance for each species, rather than assuming a single shared value for this parameter. For example, one could draw each species’ value from a shared distribution in a hierarchical model. This is likely to require a large number of individual measurements, for at least some of the species, but may be helpful in the analysis of traits that show widely differing levels of variation within different species, such as leaf trait variation across light environments [46].

## Conclusions

Why should researchers interested in performing phylogenetic comparative analyses choose to use our Bayesian methods over traditional frequentist methods? As we have demonstrated, Bayesian methods allow a lot of flexibility in the type of models that can be fitted, and Bayesian statistics provides a natural way of incorporating identified sources of uncertainty through the use of prior distributions. A central problem for frequentist phylogenetic comparative models has been that the regression estimators assume that the phylogeny is known without error. Although several authors have proposed methods to deal with phylogenetic uncertainty, few have become accessible to biologists through software applications (but see [47]), and a clear interpretation in terms of probability distributions is often lacking. Here we have shown that phylogenetic uncertainty can be readily incorporated in the estimation of linear model parameters, in freely available Bayesian software. This enabled us to drawn conclusions that do not overestimate confidence in our results, and allowed the calculation of the full posterior marginal distribution of the regression model parameters. We have shown that Bayesian methods usually out-perform their frequentist counterparts under conditions of phylogenetic uncertainty (Figure 8 and Table 1, and perform approximately as well as frequentist methods under ideal conditions (when the phylogeny is known).

In this study, we have concentrated on providing models that can be easily understood in the BUGS model programming language, and implemented using the user-friendly OpenBUGS program. We believe that most biologists who are new to Bayesian modelling will probably use this program (or a similar BUGS system, such as WinBUGS or JAGS). These programs have been designed for extreme flexibility in the types of models that can be fitted. However, this flexibility can be traded off against the speed of computation, compared to software that is more constrained in the types of models that can be fitted. One example of this is Hadfield’s MCMCglmm for R [48,67]. MCMCglmm can be forty times faster than OpenBUGS at fitting Generalised Linear Mixed-effects Models (GLMMs) [48]. We think there is room for both approaches, and the particular software environment used will probably depend on the inclination, experience and skills of the researcher, as well as the form of the particular problem at hand. Currently, MCMCglmm is constrained to fit only GLMMs, although a wide variety of commonly used models in biology fall into this category. However, OpenBUGS and JAGS can fit a much larger array of models. Also, OpenBUGS and JAGS are still under development and new features of these programs may increase their computation speed. For example, the latest version of JAGS (version 3.1.0) offers block updating of parameters in Generalised Linear Models (GLMs), similar to MCMCglmm [49].

While this study is based on the ideas of [26] in using Bayesian inference for incorporating phylogenetic uncertainty through a prior distribution, it differs in some important methodological aspects. Huelsenbeck and Rannala have developed a method which estimates the phylogeny and the comparative analysis regression simultaneously, whereas we assumed that the phylogeny was estimated independently, before modelling any relations among traits. This assumed independence means that any data sets used in phylogeny estimation cannot also be used in a comparative analysis. This will not be an issue where phylogeny estimation is based on DNA sequence data. The complex model of Huelsenbeck and Rannala requires the construction of a particular and complete MCMC algorithm, and this may be prohibitive for most researchers. We think that using BUGS syntax makes the methodology more accessible to a wider range of users and is more portable. The disconnection between phylogeny estimation and regression fitting in our approach is a departure from the methods of [26], but we believe it to be more practical. However, one has to be careful about the quality of the empirical distribution of the trees before using it for comparative analysis: using a badly estimated prior might be counter-productive. However, we think that the Bayesian framework is a very suitable tool for modelling complex and uncertain evolutionary data. Many researchers can use these tools, and since Bayesian methods are being used widely to infer phylogeny [50], posterior distributions of trees will become more commonly available for use as priors in comparative studies, e.g. [51].

Finally, we wish to emphasise the importance of model checking. Bayesian methods have been adopted enthusiastically by many researchers, but in promoting Bayesian methods, model checking is often overlooked, e.g. [32]. Bayesian methods are not a panacea for poor modelling practice [52], and care needs to be taken, as with any other kind of data analysis. Further model evaluation can be conducted by testing the sensitivity of the results to various different prior distributions [53].

## Methods

### Notation

Here, and for the rest of the paper, we use the following notation : *N* and *K* are respectively the number of species and the number of trees; $\theta =(\beta_{0},\beta_{1},\ldots ,\sigma ,\ldots )$ is the vector containing the parameters to be estimated. $\beta =(\beta_{0},\beta_{1},\ldots )$ is the vector of linear regression parameters. *Y * is a data vector of length *N* and *X* is a design matrix containing predictors for the linear regression, so that $E(y_{i}{|}_{X,\theta })=\beta_{0}x_{i,0}+\beta_{1}x_{i,1}+\ldots$ and *Σ*is a scaled variance-covariance matrix calculated from an ultrametric tree. *Σ* should be scaled to a height of one instead of being scaled to tree maximum branch length, because units of branch length may be meaningful for the phylogeny (e.g. millions of years, number of mutations...) but they are not related to the units of the trait data, and so relative lengths should be used. In this way, *σ*^2^ can be directly interpreted as the residual variance and *Σ* as a correlation structure. Other notation will be specified when needed. The distribution of *Σ* can be a computed distribution from Bayesian phylogenetic software (e.g. BEAST [35] or MrBayes [36]). In a general way, we will write:

(5) Σ∼π(ξ)

where *π* represents any relevant distribution with parameters *ξ*.

### Linear regression model

In order to illustrate the practical nature of our methods, we first give a simple example. One classic model for comparative analysis is a linear regression across a multispecies data set. To construct it, we used a multivariate normal for the likelihood and conjugate priors. The model can be specified as follows:

(6) $$\begin{array}{ll} Y|X,\beta ,\sigma ,\Sigma & ∼N(\mathrm{X\beta},\sigma^{2}\Sigma )\\ \beta \;\; & ∼\;\;N(0,10^{6})\;\\ \sigma^{-2}\;\; & ∼\;\;\Gamma (\theta ,\theta )\;\\ \Sigma \;\; & ∼\;\;\pi (\xi )\; \end{array}$$

The priors on the components of *β*are the usual non-informative conjugate univariate normal priors [33, p.578-583]. The Gamma prior (labelled Γ) on *σ*^−2 ^ is weakly informative for small variance [54], depending on the value of *ε*, but its conjugacy with the multivariate normal seems to help in avoiding the problem of autocorrelation in successive samples from the Markov chain Monte Carlo. This model is quick to converge and usually shows negligible autocorrelation.

### Measurement error model accounting for intraspecific variation

Comparative analyses frequently represent each species by a single value, such as a mean estimated from a small sample of individuals. Often, the intraspecific variance in trait values is not considered. Such variance can arise from sources including meaningful biological variation among individuals, inaccuracies of measurement, or poor sampling. Analyses that do not consider such "measurement error" may lead to biases or inaccuracies in evolutionary inferences [55].

Here we develop a model for the relationship between two traits across species, and incorporate variation across individuals within species, by using measurements from multiple individuals per species. The forms of intraspecific variation that this model can incorporate are: *(i)* natural variation across individuals that is not correlated between the two traits, and/or *(ii)* the observer’s ’measurement errors’ sensu stricto. Therefore this model does not incorporate phenotypic covariance *within* species (i.e. the situation where the values of each trait are correlated across individuals within the species), though it does incorporate phylogenetic covariance of the traits across species.

Here we focus on the situation where one measurement was taken per individual, and hence we treat natural variation and measurement error *sensu stricto* together. However, it would be possible to model these two variance components separately, if multiple observations (measurements) were made on each individual, for multiple individuals per species.

We take several *individual measurements*, and assume these to be normally distributed around an unknown species mean (which we call the *species level value*). The evolutionary relationship between two traits is modelled as a linear relation between the unobserved species mean values. This model is therefore a form of measurement error model [56]. We denote the *individual measurements* for each trait by the *N*×*n* matrices *W * and *V *, where *N* is again the number of species and *n* is the number of observations per species. The *species level* variables are defined as the (unobserved) corresponding vectors *X* and *Y *. The individual variances of trait measurements are assumed to be constant across species and are denoted respectively $\sigma_{W}^{2}$ and $\sigma_{V}^{2}$. The residual standard deviation of the relation between *X* and *Y * will be designated *σ*_*R*_. Note that both *X* and *Y * have the same phylogenetic correlation structure (*Σ*), and we assume that the measurements are uncorrelated within individuals. The model can then be written as follows:

(7) $$\begin{array}{ll} \;W_{\text{ni}}|X_{n},\sigma_{W}\;\; & ∼\;\;N(X_{n},\sigma_{W}^{2})\;\\ \;V_{\mathrm{ni}}|Y_{n},\sigma_{V}\;\; & ∼\;\;N(Y_{n},\sigma_{V}^{2})\;\\ \;X|\mu_{0},\sigma_{0},\Sigma \;\; & ∼\;\;N(\mu_{0},\sigma_{0}^{2}\;\Sigma )\;\\ \;Y|X,\beta ,\sigma_{R},\Sigma \;\; & ∼\;\;N(\mathrm{X\beta},\sigma_{R}^{2}\;\Sigma )\;\\ \;\beta \;\; & ∼\;\;N(0,10^{6})\;\\ \sigma_{W}^{-2},\sigma_{V}^{-2},\sigma_{R}^{-2}\;\; & ∼\;\;\Gamma (\theta ,\theta )\;\\ \;\Sigma & ∼\;\;\pi (\xi ) \end{array}$$

After initial experimentation, we found that a weakly informative prior on the species level *X* with sensible parameters that cover a range thought to be biologically possible (such as *μ*_0_=0.5 and *σ*_0_=0.5 for a trait known to be between zero and one) enabled the model to return more stable estimates for *X*, especially when some species have only a small number of individual measurements. Autocorrelation of the values sampled by the MCMC chain can become important for some datasets. In this case, we found that plausible starting values and having a longer burn-in helped to ensure that the model converged on its equilibrium distribution, since autocorrelation becomes only a minor issue when convergence is reached. In OpenBUGS, this model failed to run for data sets with a large number of species *N*, probably because of memory issues. This problem was not encountered in JAGS, and does not appear to derive from theoretical problems in the estimation.

### Phylogenetic signal model

It is often of interest to quantify the strength of phylogenetic signal [57] in the values of a trait across present-day species and to compare this strength among traits or for trees of different lineages. For this purpose, we constructed a model to estimate Pagel’s *λ*in the response trait *Y *[37,58] simultaneously with the estimation of linear regression parameters for the relation between *X* and *Y *. This model has been treated in a Bayesian context by [59,60]. Unlike the Measurement Error model, we do not assume any phylogenetic signal in *X*[61]. In this model, *λ* is a coefficient that multiplies the off-diagonal elements of the variance-covariance matrix *Σ*. A *λ* close to zero implies that the phylogenetic signal in the data is low, suggesting independence in the error structure of the data points, whereas a *λ* close to one suggests a good agreement with the Brownian Motion evolution model and thus suggests correlation in the error structure. Since our variance-covariance matrices (indeed correlation matrices) have all diagonal elements equal to 1, we can incorporate *λ* into the matrix using this simple calculation:

(8) $$\Sigma_{\lambda }=\lambda \;\Sigma +(1-\lambda )\;I$$

where **I** is the identity matrix. The model can then be written as:

(9) $$\begin{array}{ll} \;Y|X,\beta ,\sigma ,\Sigma ,\lambda \;\; & ∼\;\;N(\mathrm{X\beta},\sigma^{2}\Sigma_{\lambda })\;\\ \;\beta \;\; & ∼\;\;N(0,10^{6})\;\\ \;\sigma^{-2}\;\; & ∼\;\;\Gamma (\varepsilon ,\varepsilon )\;\\ \;\lambda \;\; & ∼\;\;U(0,1)\;\\ \;\Sigma \;\; & ∼\;\;\pi (\xi )\; \end{array}$$

This model estimates the regression coefficients *β* as well as *λ*, which has a uniform prior (labelled U). In our experiments, the MCMC sample for *β* showed a small degree of autocorrelation, but converged quickly. Using JAGS, some data sets with small values for *X* or *Y * showed a very large autocorrelation on the estimates for *λ*. This issue can be avoided by scaling data values (by a factor 10 for example), or equivalently, use a different prior for *σ*. We found that simulated data sets with fewer than 20 species have very low power to detect phylogenetic signal (as found in [57]). For simulated data (for which *λ* should be estimated as unity), *N* = 5 led to an estimate of *λ* of 0.40 with standard deviation of 0.27, whereas for *N* = 10, this became 0.74 (0.24) and for *N* = 25, we obtained *λ*=0.90(0.10).

### Model checking

A fundamental part of statistical modelling is checking the goodness-of-fit of the model to the data. That is, does the model adequately capture the properties of the data? This procedure is called "posterior checking" in the Bayesian framework [62,63]. Of course, the first checks concern the relevance of the estimates and their distribution. To assess the performance of each model in capturing the properties of the data, we also performed posterior checks [62,63] based on the posterior predictive distributions (checking agreement between the observed data, and simulated replicates of the data, generated by simulation from the selected model). This is a method for assessing the discrepancy between the model and the data, based on the distribution of a discrepancy test statistic *D*(*yθ*). Since we are interested in the overall goodness-of-fit of the model, we used a function related to the *χ*^2^function suggested by [63]. However, in our case, the points are not independent, as they are related through a correlation structure. Thus, instead of using the *standardised* residuals to define the usual *χ*^2^, we will use the *normalised* residuals, defined by [64]:

(10) $$\varepsilon =\sigma^{-1}{\left(\Sigma^{-\frac{1}{2}}\right)}^{T}\times (Y-E(Y|\theta ))$$

where *T* is the canonical symbol for "transpose of the matrix". *Y * is a column vector, so this matrix multiplication returns a column vector of residuals as a result. Our discrepancy function can then simply be written:

(11) $$D(y,\theta )=\underset{i}{\sum }{\left(\varepsilon_{i}\right)}^{2}$$

The essence of the posterior predictive check is to compute this distribution for hypothetical replicates of the data *Y*^*rep*^ and see if the value for the data *Y * is in agreement with this distribution. In order to simulate *Y*^*rep*^, it is necessary to integrate over all the possible parameter values. One solution is to draw *L* parameter values directly from the MCMC posterior samples. We can then calculate:

(12) $$\forall \;l\in 1,\ldots ,L:\;D(y_{l}^{\text{rep}},\theta_{l})-D(y,\theta_{l})$$

and compute the *ppp-value* (for posterior predictive *p-value*) as: $P(D(y_{l}^{\text{rep}},\theta_{l})-D(y,\theta_{l})>0)$ (see example in Figure 3). If we are interested in other discrepancy measures *D*^∗^(*Y*|*θ*) (for example *max*(*Y *), *mean*(*Y *), or *sd*(*Y *)), we can use the same draws to calculate them, allowing us to check different parts of the model at the same time. One other interesting source of information is to compute the *species ppp-value* (*sppp-value*) for each species or taxon, which we define as follows:

(13) $$P({\left(\varepsilon_{i}^{\text{rep}}\right)}^{2}-{\left(\varepsilon_{i}\right)}^{2}>0).$$

Discrepancy values are used to compare the dispersion of the replicates to the dispersion of the data and detect potential outliers or consistent over- and underdispersion (see examples in Figures 3 and 4). For example, ppp-values close to zero indicate that most of the replicated datasets were less extreme than the observed datasets, and hence the model shows less discrepancy from predicted values, compared to the real data. A high ppp-value indicates that the model generates replicate datasets that show consistently greater discrepancy from predicted values than does the observed dataset. For sppp, a high value indicates consistent overdispersion within the replicate datasets.

For the Measurement Error model, we split the posterior checking into two parts. We assessed the estimates for the parameters of the linear relation, and for the species-level values *X* and *Y *, and we also assessed the distribution of the individual measurements (within species). Assessing the accuracy of the species level value is difficult, because we have no theoretical expectation for these values. However, we can compare the estimates and the mean of the individual measurements or we can compute a ppp-value using $D(Y|\theta )=\underset{i}{\sum }(Y_{i}-\bar{V_{i}})$as a discrepancy statistic, $\bar{V_{i}}$ being the mean of the individual measurements for the species *i*. Afterward, the residuals from the linear relationship residuals were checked using the previous method (seeing $\bar{V}$ as the data "species value"). We checked the individual measurements for each species using standardised residuals to compute a sppp-value (in contrast to the normalised residuals used above, because we view the measurements as independent within a species, rather than correlated).

The ppp-value is not the probability of the model being true. Rather it is the probability of observing more extreme data than the current data set, given the model assumptions, the posterior distribution of parameters and the discrepancy statistic. Therefore, our use of ppp-values is solely to assess how "surprising" the data appear to be under the model assumptions and the parameters estimates. If the ppp-value is very extreme (close to zero or one), this alerts us to possible structural problems in the model, since it means that the distribution of data simulated from the model differs from the data we actually observed for a particular aspect of the model (distribution of residuals, mean, etc.). This can help to identify aspects of the model that are failing to represent the data adequately and should be altered (see an example in our Real Data analysis with Phylogenetic Signal model). Unlike classical p-values, the Bayesian ppp-values are not necessarily uniformly distributed under the null hypothesis and should not be compared across models or be used to set a permissible type I error rate (false rejection of the model, [65]): there is no "critical value" such as 0.05 with ppp-values. For a detailed explanation about the interpretation of ppp-values, see [63].

If the interest is in comparing *β*_*i*_ to a particular value, you can simply give the posterior probability that *β*_*i*_ falls in any particular range of values. For example, you might want to know the probability of values less than zero, or greater than zero, or within a certain distance of zero. Bayesian inference enables us to make a direct statement about this probability, rather than accepting or rejecting a point hypothesis with an assumed significance level. The probability is equal to the proportion of the area under the probability density function that falls in a particular range. For example, if we were interested in whether *β*_*i*_ was greater than or less than zero, and the posterior distribution had only 1% of its area in a lower tail extending into negative numbers, then we would conclude that the probability that *β*_*i*_ is less than zero, given the data, is 0.01. By the same finding, the probability that *β*_*i*_ is positive, is 99%.

### Implementation of models and data analysis

The general aim for our models is to estimate the posterior distribution of parameters of a model where the data are correlated through a phylogenetic relationship for which we have a prior distribution. The two main assumptions of our models are *(i)* that the phylogenetic trees are ultrametric, so that the correlation matrix is proportional to the variance-covariance matrix and *(ii)* that evolution can be modelled by a Brownian Motion (BM) process. These assumptions are common in the comparative analysis literature, for example [66], but can be relaxed in some situations, e.g. [57].

We used the statistics software R [67] for data handling in association with OpenBUGS 3.0.3 [68] for Markov Chain Monte Carlo (MCMC) computation. MCMC algorithms use iterative draws to sample from an unknown target distribution, and thereby learn about its properties. In our case, the target is the marginal posterior distribution of parameters in our model. In each step, a draw is made of a new value for one parameter, conditional on the data and current values of all the other parameters in the model. Over a sufficiently large number of iterations, the algorithm converges to the marginal distribution of the parameters, see [33,69]. The samples following this initial period of convergence (the ’burn in’) can be used for inference on the parameters. The three kinds of algorithm currently used by OpenBUGS are the Gibbs sampler [70,71], the Metropolis-Hastings algorithm [72,73] and the slice sampler [74]. After prior sensitivity analysis, we decided to use an inverse-Gamma(1,1) prior distribution for variance components. This prior helped to avoid autocorrelation and had little influence on our results (see Figure 8 for example). However, we do not recommend such an informative prior without preliminary analysis of prior sensitivity.

MCMC algorithms sometimes exhibit excessive autocorrelation among successive values in the chain, leading to inefficient sampling of the full parameter space if the dependence among samples extends for more than a few iterations. If the autocorrelation is high for a parameter, it may be necessary to let the simulation run longer and take a subsample of the MCMC output. We discuss autocorrelation issues for each model, below, along with other features of their application.

We report ’ppp-values’ (posterior predictive p-values) as an indicator of probabilities of the observed data, under the best-fit model; and ’sppp-values’ (species ppp-values, for each species) as an indicator of the under or overdispersion in replicate datasets generated under the best-fit model (see "Model checking" section).

## Authors’ contributions

SPB Conceived the study, wrote some of the BUGS code, and contributed to writing the manuscript. PdV wrote most of the BUGS code, conducted all analyses, and contributed to writing the manuscript. JAW conceived the Measurement Error model and assisted in analyses, and JAW and RDE both contributed data and helped write the manuscript. All authors have read and approved the final manuscript.

## Supplementary Material

Additional file 1 **Appendix.** BUGS code for the models. Click here for file
