# Supplementary material for: Bayesian models for comparative analysis integrating phylogenetic uncertainty
Source: BMC Evol Biol. 2012 Jun 28;12:102. doi: 10.1186/1471-2148-12-102 (PMC3582467; doi:10.1186/1471-2148-12-102)
Supplement: Additional file 1 — Appendix. BUGS code for the models. [file 1471-2148-12-102-S1.pdf]

Appendix : BUGS code for the models

Linear Regression Model

This is the OpenBUGS code for the LR model with an empirical distribution based on a set of estimated trees. In our models, we called β_0 `alpha` and β_1 `beta`. `Nspec` stands for N and `Ntree` for K. `invA` is an array of dimension $N \times N \times K$ in which the element `invA[,,i]` is the inverse of the variance-covariance matrix for tree i:

```
model {
#Linear regression and multivariate normal likelihood
  for (i in 1:Nspec) {
    mu[i] <- alpha+beta*X[i]
  }
  Y[1:Nspec] ~ dmnorm(mu[],TAU[,])

#Priors
  alpha ~ dnorm(0,1.0E-06)
  beta ~ dnorm(0,1.0E-06)
  tau ~ dgamma(1,1)
  sigma <- 1/sqrt(tau)

#Equal vector of probability for tree sampling
  for (k in 1:Ntree) {
    p[k] <- 1/Ntree
  }

#Tree sampling and variance-covariance matrix construction
  K ~ dcat(p[])
  for (i in 1:Nspec) {
    for (j in 1:Nspec) {
      TAU[i,j] <- tau*invA[i,j,K]
    }
  }
}
```

In JAGS, one can avoid defining `TAU` in a loop by directly incorporating its value `tau*invA[,,K]` in the likelihood specification. If one wants to use the Wishart prior, one can use the following line instead of the two last parts:

```
  Tau[1:Nspec,1:Nspec] ~ dwish(SIGMA[,],nu)
```

where `SIGMA` stands for $(N+2)\Sigma_0$ (BUGS has a non conventional specification) and `nu` is $N+2$.

Measurement Error model

OpenBUGS code for ME model. `NrepW` and `NrepV` are vectors containing the number of individual measurements for each species:

```
model {
#Multivariate normal distribution of the specific level value
  Y[1:Nspec] ~ dmnorm(meany[], TAUY[,])
  X[1:Nspec] ~ dmnorm(meanx[], TAUX[,])

  for (i in 1:Nspec) {
#Prior mean of the specific level X (to be changed according to data set)
    meanx[i] <- -10
#Linear relation between Y and X
    meany[i] <- alpha + beta * X[i]
#Replicates normal independent distributions
    for (j in 1:NrepW[i]) {
      W[i,j] ~ dnorm(X[i], tauw)
    }
    for (l in 1:NrepV[i]) {
      V[i,l] ~ dnorm(Y[i], tauv)
    }
  }

#Priors
  tauw ~ dgamma(1,1)
  tauv ~ dgamma(1,1)
  taur ~ dgamma(1,1)
  alpha ~ dnorm(0, 1.0E-6)
  beta ~ dnorm(0, 1.0E-6)

  sigmar <- 1/sqrt(taur)
  sigmaw <- 1/sqrt(tauw)
  sigmav <- 1/sqrt(tauv)

#Empirical distribution (taux is fixed a priori,)
  for (k in 1:Ntree) {
    p[k] <- 1/Ntree
  }
  K ~ dcat(p[])
  for (i in 1:Nspec) {
    for (j in 1:Nspec) {
#Prior precision to be changed according to data set
      TAUX[i,j] <- 0.01*invA[i,j,K]
      TAUY[i,j] <- taur*invA[i,j,K]
    }
  }
}
```

Pagel's λ model

OpenBUGS code for PL model:

```
model  {
#Linear regression and multivariate normal likelihood
  for (i in 1:Nspec) {
    mu[i] <- alpha+beta*X[i]
  }
  Y[1:Nspec]~dmnorm(mu[],TAU[,])

#Priors
  alpha ~ dnorm(0,1.0E-06)
  beta ~ dnorm(0,1.0E-06)
  lambda ~ dunif(0,1)
  tau ~ dgamma(1,1)
  sigma <- 1/sqrt(tau)

#Tree sampling and lambda computation
  for (k in 1:Ntree) {
    p[k] <- 1/Ntree
  }
  K~dcat(p[])
  for (i in 1:Nspec) {
    for (j in 1:Nspec) {
#LAMBDA is a matrix with off-diagonal lambda value and 1 in the diagonal
      LAMBDA[i,j] <- 1 + (lambda-1)*(1-equals(i,j))
      TAU[i,j] <- tau*LAMBDA[i,j]*invA[i,j,K]
    }
  }
}
```

IN JAGS, the computation of λ can be simplified (with `ID` being the identity matrix):

```
  Mlam <- lambda*A[,,K]+(1-lambda)*ID
  TAU <- tau*inverse(Mlam)
```

In that case, `A` is the array of the variance-covariance matrices, not their inverses.
